# Avian haemosporidian parasite prevalence and diversity in two populations of the American kestrel (*Falco sparverius*)

**DOI:** 10.1007/s00436-025-08509-w

**Published:** 2025-06-09

**Authors:** Laura Kwasnoski, Jordan Brown, Jessica Taylor, Jesse L. Watson, Dave Oleyar, Vincenzo A. Ellis

**Affiliations:** 1 Department of Entomology and Wildlife Ecology, University of Delaware, Newark, DE USA; 2 Delaware Department of Natural Resources and Environmental Control, Division of Fish and Wildlife, Dover, DE USA; 3 HawkWatch International, Salt Lake City, UT USA

**Keywords:** Avian malaria, *Haemoproteus*, *Leucocytozoon*, Pathogens, Raptors

## Abstract

Parasite communities vary among host species and across space. However, little is known about differences in parasite communities between geographically and genetically distinct populations of the same host species. American kestrels (*Falco sparverius*) are small falcons with regionally distinct genetic populations across North America. We sampled kestrels from Delaware and Utah for avian haemosporidian parasites (genera: *Haemoproteus*, *Plasmodium*, and *Leucocytozoon*) and used molecular barcoding of the parasite cytochrome *b* gene (cyt *b*) to quantify parasite genetic lineage diversity. We identified four lineages of *Haemoproteus* parasites and one *Leucocytozoon* lineage infecting kestrels. A comparison with previous studies suggests that most of these lineages are largely restricted to kestrels. We found similar infection prevalence and lineage composition between the sites. All kestrels sampled in Utah were adults (i.e., sampled after hatch year), but in Delaware, we found adult birds had a higher infection prevalence than juveniles (i.e., hatch-year birds). Despite harboring largely the same parasite lineages, kestrels are unlikely to disperse between Utah and Delaware. The similarity in parasite lineages in the two kestrel populations could be due to a number of factors including broadly distributed vector species (of which little is known), movement of alternative and undetected host species, or transmission during migration or on overwintering grounds. Alternatively, the cyt *b* gene might not capture recent genetic differentiation among the parasites. Future studies should explore these various possibilities to understand the mechanisms underpinning parasite distributions across genetically structured host populations.

## Introduction

Parasite communities can change across a landscape due to dispersal limitations of the parasites and/or turnover in the presence or abundance of host species. For example, the species and prevalence of ectoparasites (fleas and mites) of small mammals have been found to change across space and correlate with changes in host species distributions and climate variation (Krasnov et al. 2020). Landscape topography may be another impediment to parasite dispersal (Poulin and Krasnov 2010), unless parasites are highly mobile (Eriksson et al. 2019). Similarly, vector-transmitted parasites may disperse more readily than their hosts (Eriksson et al. 2019), particularly if their vectors are highly mobile. Such parasite dispersal could lead to hosts with distinct population genetic structure being infected by a single genetic population of parasites across their range. More parasite surveys across the ranges of dispersal limited host species are needed to begin to create generalizations about parasite dispersal and its consequences for hosts.

Avian haemosporidian parasites are ideal for investigating parasite distributions across host populations. Avian haemosporidians are well-studied, vector-transmitted, and globally distributed with the exception of Antarctica (Garnham 1966; Valkiūnas 2004). These parasites are generally classified into the taxonomic genera *Haemoproteus, Plasmodium*, and *Leucocytozoon.* Parasites from each genus are transmitted by different dipteran vectors: *Haemoproteus* is vectored by biting midges (family: Ceratopogonidae) or louse flies (family: Hippoboscidae), *Plasmodium* is vectored by mosquitoes (family: Culicidae), and *Leucocytozoon* is vectored by black flies (family: Simuliidae) (Valkiūnas 2004; Atkinson et al. 2008; Santiago‐Alarcon et al. 2012). Infections in birds can be determined by microscopic examination of blood smears (Valkiūnas 2004) and/or molecular screening. Molecular barcoding of the parasite’s cytochrome *b* gene (cyt *b*) is commonly used to identify the genetic parasite lineage (Hellgren et al. 2004); a single nucleotide difference in the sequenced region of cyt *b* is the criterion typically used to define a unique lineage (Bensch et al. 2009). Avian haemosporidians range from specialists to generalists (Ellis and Bensch 2018) and some can be found throughout the ranges of their host species (Ellis et al. 2015; Huang et al. 2025). When statistically controlling for host species turnover, avian haemosporidian lineage turnover appears unrelated to geographic distance (Ellis et al. 2015) and infections of the same haemosporidian lineages have been found across a species’ range (Fallon et al. 2006; Szöllősi et al. 2011), even spanning continents (Ellis et al. 2019). Therefore, one can hypothesize that the composition of haemosporidian lineages is likely to be similar among genetically distinct host populations; however, more evidence is needed, particularly from under-sampled host populations like raptors.

American kestrels (*Falco sparverius*) are a small falcon species widely distributed across the Americas with 17 recognized sub-species, two of which are found in the USA (Ferguson-Lees and Christie 2001; Gill et al. 2024). Among kestrels in the USA and Canada, there are five genetically distinct populations (“eastern,” “western,” “Texas,” “Florida,” and “Alaska;” Ruegg et al. 2021). Kestrels are partial migrants that display differential intraspecific migration, with birds at more temperate breeding-season latitudes migrating shorter distances or not migrating at all compared to kestrels from higher latitudes (Goodrich and Smith 2008; Goodrich et al. 2012; Heath et al. 2012). Efforts to track migrating kestrels are ongoing (Crandall and Craighead 2019; Hunt et al. 2023). Avian haemosporidian parasites have been surveyed in kestrels from several parts of their range. In California, wild kestrels were sampled during the breeding season and 37% were infected with *Leucocytozoon* (Walther et al. 2016). In Texas, a *Haemoproteus* lineage (FASPA04) was identified infecting kestrels (Keith et al. 2022). *Haemoproteus* parasites have also been found in kestrels sampled from the southern Chihuahua Desert of central Mexico (Tinajero et al. 2019) and the Baja California peninsula (Frixione and Rodríguez-Estrella 2023). Given that the range-wide genetic population structure of North American kestrels has been established (Ruegg et al. 2021), kestrels are an ideal host species to investigate parasite turnover across their range.

Here we sampled avian haemosporidians in wild kestrels from Utah and Delaware. These two locations likely represent the genetically distinct “western” and “eastern” kestrel populations of North America, respectively (Ruegg et al. 2021). We used a multiplex PCR to identify parasite infections (Ciloglu et al. 2019) and sequenced cyt *b* to identify parasite lineages in infected birds (Bensch et al. 2009). We then investigated whether the two host populations were infected by the same or different parasite lineages to better understand how parasites are distributed across host populations. We also investigated the host specificity of the lineages we identified by comparing our results with the distributions of those lineages identified in previous studies.

## Methods

### Study sites and sample collection

American kestrels were sampled at two sites across North America in collaboration with one non-profit and one state agency. Locations were chosen from two geographically and likely genetically distinct populations of American kestrels—the “eastern” and “western” groups represented by Delaware and Utah, respectively (Ruegg et al. 2021).

From May to June 2024, 21 wild kestrels were captured and had small blood samples taken by HawkWatch International (HWI) biologists in the greater metropolitan area of Salt Lake City, Utah (centroid coordinates of sample locations: 40.76111935, − 111.9850279). HWI spearheads a long-term kestrel demography study monitoring over 500 nest boxes and approximately 130 kestrel pairs during the kestrel breeding season each year. All the kestrels sampled by HWI were adults (i.e., after hatch-year birds).

From March 2023 to June 2024, 35 wild kestrels were sampled across the state of Delaware (centroid coordinates of sample locations: 39.6152001, − 75.60631524) by state biologists and trained professionals at Delaware’s Department of Natural Resources and Environmental Control (DNREC) Division of Fish and Wildlife. DNREC maintains and regularly monitors approximately 90 nest boxes throughout Delaware during the kestrel breeding season (March to July) as well as opportunistically sampling non-breeding birds throughout the year.

Following permits and protocols established within each organization, wild kestrels were safely captured using methods including bal-chatri traps, hand-grabbing from nest boxes, and mist net trapping (with and without animatronic owl decoy lures). Each bird was sexed, aged (juveniles or hatch year “HY” birds including nestlings; adult or and after hatch year “AHY” birds), fitted with a federally issued metal leg band, and measured, before *ca.* 50–70 μl of whole blood was collected from their brachial, femoral, or jugular veins. Kestrels were then released or returned to nest boxes. Blood samples were immediately added to 1.5 μl microcentrifuge tubes pre-loaded with RNAlater Stabilization Solution (ThermoFisher Scientific, Waltham, MA, USA) and frozen at − 20 ℃ within 24 h of sampling. A limitation of this study is that blood smears were not made and therefore individuals could not be examined for infection through microscopy.

### Molecular screening and DNA sequencing

All kestrel blood samples were sent to the University of Delaware (Newark, DE) for molecular analysis. We extracted DNA from the blood samples using a DNeasy Blood and Tissue Kit (Qiagen, San Francisco, CA, USA) and quantified DNA concentrations using a Nanodrop One Spectrophotometer (ThermoFisher Scientific, Waltham, MA, USA). DNA samples were diluted with ultrapure water to a concentration of *ca.* 25 ng/µl.

We tested each sample for *Haemoproteus*, *Plasmodium*, and *Leucocytozoon* infection using a multiplex polymerase chain reaction (PCR; Ciloglu et al. 2019). The multiplex PCR uses the following primers: HMF 5′-ATTGGATGTCAATTACCACAATC-3′and HMR 5′-GGGAAGTTTATCCAGGAAGTT-3′ for *Haemoproteus*, PMF 5′-CCTCACGAGTCGATCAGG-3′ and PMR 5′-GGAAACCGGCGCTAC-3′ for *Plasmodium*, and LMF 5′-TGGAACAATAATTGSATTATTTACAYT-3′ and LMR 5′-AACATATCATATTCCATCCATTTAGATTA-3′ for *Leucocytozoon.* In addition to 0.2 µl of each primer (at 10 µM concentration) and 2 µl of extracted DNA (25 ng/µl), we combined 5.0 µl of Qiagen 2X Master Mix (Qiagen, San Francisco, CA, USA) and 1.8 µl ultrapure water into reaction wells. Reagents were mixed, centrifuged, and run on a MiniAmp Plus Thermal Cycler (ThermoFisher Scientific, Waltham, MA, USA) with the following thermal profile: 15-min initial denaturation (95 °C); 35 cycles of 30-s denaturation (94 °C), 90-s annealing (59 °C) and 30-s extension (72 °C); 10-min final extension (72 °C). We ran 5 µl of multiplex PCR product from each sample mixed with 1 µl of 6X TriTrack loading dye (ThermoFisher Scientific, Waltham, MA, USA) through 2% agarose gels stained with GelRed (Biotium Inc., Fremont, CA, USA) and visualized the results with a GelDoc Go (Bio-Rad, Hercules, CA, USA). Bands from each sample were compared to bands from a triple-infection positive control (i.e., a mix of DNA from birds infected with all three parasite genera) to confirm infection. We also included a 100 bp DNA ladder to verify amplified fragment lengths in each gel and an ultrapure water negative control in all PCRs and gels.

We amplified the molecular barcoding region of cyt *b* from samples infected with haemosporidians as demonstrated by the multiplex PCR. To amplify cyt *b*, we used a standard nested PCR (Hellgren et al. 2004). This two-step process included an initial PCR, where we combined 2 µl of extracted DNA (25 ng/µl) with 12.5 µl DreamTaq Master Mix 2X (ThermoFisher Scientific, Waltham, MA, USA), 9.5 µl ultrapure water, and 1 µl of each 10 µM primer (forward: HaemNFI 5′-CATATATTAAGAGAAITATGGAG-3′; reverse: HaemNR3 5′-ATAGAAAGATAAGAAATACCATTC-3′) and amplified DNA with the following thermal profile: 2-min initial denaturation (95 °C); 20 cycles of 30-s denaturation (95 °C), 30-s annealing (50 °C) and 60-s extension (72 °C); 10-min final extension (72 °C). Then, we then ran a second PCR reaction for *Haemoproteus*/*Plasmodium* or *Leucocytozoon* as described in Hellgren et al. (2004). This included combining 1 µl of PCR product from the first reaction with 12.5 µl of DreamTaq Master Mix 2X, 8.5 µl ultrapure water, and 1 µl of each primer (10 µM concentration; *Haemoproteus/Plasmodium* primers: forward HaemF 5′-ATGGTGCTTTCGATATATGCATG-3′, reverse HaemR2 5′-GCATTATCTGGATGTGATAATGGT-3′; *Leucocytozoon* primers: forward HaemFL 5′-ATGGTGTTTTAGATACTTACATT-3′, reverse HaemR2L 5′-CATTATCTGGATGAGATAATGGIGC-3′). The sample was then run through the following PCR thermal profile: 2-min initial denaturation (95 °C); 35 cycles of 30-s denaturation (95 °C), 30-s annealing (50 °C) and 60-s extension (72 °C); 10-min final extension (72 °C). To confirm successful amplification, we followed the same gel and visualization protocol as described previously. We used a negative control (ultrapure water) and positive control (DNA from a bird with a previously sequenced infection) in all reactions; the second reaction used a new negative control (ultrapure water) and the negative control from the first reaction (a “nested negative”).

We sent PCR products from the second (“nested”) reaction to Azenta Life Sciences (South Plainfield, NJ, USA) for exo-sap purification and Sanger sequencing in both the forward and reverse directions. We analyzed the Sanger sequences in Geneious R.11.1.5 (https://www.geneious.com) and used BLAST to compare the sequences to the MalAvi database (Bensch et al. 2009). All parasite lineage data from this study have been submitted to MalAvi.

### Statistical analyses

To test for differences in parasite lineage composition and prevalence, we used Fisher’s exact tests with the function *fisher.test()* in R v.4.4.2 (R Core Team 2024) We tested whether parasite lineage composition and prevalence differed among the two sites and whether prevalence varied with bird sex and age. We summarized and plotted our data using packages hosted in the R package tidyverse v.2.0.0 (Wickham et al. 2019).

## Results

### Similar prevalence in Utah and Delaware

We found 31.4% (11/35) of kestrels in Delaware infected with a parasite (all were *Haemoproteus*) and 47.6% (10/21) of birds from Utah infected (10 *Haemoproteus* infections, 1 *Leucocytozoon*) (Table 1). The prevalence of *Haemoproteus* did not differ significantly between the Delaware and Utah populations (odds ratio = 0.511, *P* = 0.264). Since there was only one *Leucocytozoon* infection, we did not test for a difference in the prevalence of that genus.

**Table 1:** Samples analyzed from each site. The number of birds sampled and the number of birds infected with each parasite genus (*Haemoproteus*, *Leucocytozoon*, *Plasmodium*) are reported. One bird from Utah was infected with both *Haemoproteus* and *Leucocytozoon* and was therefore reported once in the “number of *Haemoproteus* infections” column and once in the “number of *Leucocytozoon* infections” column

Sampling location	Number of samples	Number of *Haemoproteus* infections	Number of *Leucocytozoon* infections	Number of *Plasmodium* infections
Delaware, USA	35	11	0	0
Utah, USA	21	10	1	0

### Prevalence in relation to age and sex

Infection by *Haemoproteus* varied significantly by kestrel age in Delaware (odds ratio = 26.784, *P* < 0.001) with more adult birds (75%; 9/12) infected than juveniles (8.7%; 2/23). Within Delaware, the prevalence of *Haemoproteus* was lower in male (13.3%, 2/15) than female kestrels (45%, 9/20) but this difference was not statistically significant (odds ratio = 0.197, *P* = 0.069). It is important to note that the relatively low *P* value in the test comparing Delaware males and females (0.069) was influenced by the ages of the birds sampled. There were many more adult females sampled (nine; eight of which were infected, i.e., 8/9 infected) than adult males (three; one of which was infected, i.e., 1/3 infected; odds ratio = 11.094, *P* = 0.127) and there was no difference between juvenile females (1/11 infected) and males (1/12 infected), which were more evenly sampled (odds ratio = 1.095, *P* = 1). At the Utah site, *Haemoproteus* prevalence did not vary significantly by sex (log odds = 0.531, *P* = 0.659; 3/8 males and 7/13 females infected).

### Limited parasite lineage turnover between Utah and Delaware

We found four *Haemoproteus* lineages, with three of the four lineages found at both sites (Table 2). The lineage BNOW03 was found in the greatest number of kestrels (nine infections in Delaware, five infections in Utah) followed by BNOW02 (two in Delaware, two in Utah) and SALUR01 (one in Delaware, one in Utah). The fourth lineage, FASPA03 was only found in a single kestrel from Utah. Additionally, two birds from Utah had co-infections (likely multiple *Haemoproteus* lineages) that we were unable to identify to lineage (for a discussion of phasing co-infections with Sanger sequence data, see Drovetski et al. 2014 and Matthews et al. 2016). We also found one *Leucocytozoon* lineage, FASPA02, in a Utah kestrel. We found no statistical differences in the frequencies of parasite lineages between the two sites (BNOW02, odds ratio = 0.582, *P* = 0.626; BNOW03, odds ratio = 1.106, *P* = 1; FASPA02, odds ratio = 0, *P* = 0.375; FASPA03, odds ratio = 0, *P* = 0.375; SALAUR01, odds ratio = 0.594, *P* = 1; Table 2). Among the juvenile birds from Delaware, we found the lineage BNOW03 (2/23 infected), indicating that the lineage is transmitted in Delaware.

**Table 2:** Number of birds infected with each parasite lineage from each site. Parasite lineage co-infections were recorded once in each column for which they had an infection. For example, a Delaware bird infected with both BNOW02 and BNOW03 was reported once in both the “BNOW02” and “BNOW03” columns. Unknown *Haemoproteus* infections were co-infections (as determined by double peaks in the sequencing chromatogram) that we could not separate into distinct lineages. The parasite genus is reported in parentheses below the lineage name (abbreviated as follows: “*Haemo*.” = *Haemoproteus,* “*Leuco.*” = *Leucocytozoon*)

	Parasite lineages					
Sampling location	BNOW02 (*Haemo*.)	BNOW03 (*Haemo*.)	FASPA02 *(Leuco.)*	FASPA03 (*Haemo*.)	SALAUR01 (*Haemo*.)	Unknown *Haemo*. infection
Delaware, USA	2	9	0	0	1	0
Utah, USA	2	5	1	1	1	2

### Lineage distributions and host specificities

Of the four *Haemoproteus* and one *Leucocytozoon* lineages we identified, all except one had previously been found infecting kestrels within their range (Table 3). The lineage BNOW02, which we found in both Delaware and Utah kestrels, had previously only been found in an American barn owl (*Tyto furcata*; Table 3).

**Table 3:** Additional parasite lineage information from other studies that identified the haemosporidian lineages found in this study. Existing parasite lineage data were extracted from the MalAvi database. Parasite lineage genus is reported in parentheses below the lineage name (abbreviated as follows: “*Haemo*.” = *Haemoproteus*, “*Leuco.*” = *Leucocytozoon*)*.* Regional location data are provided when possible and countries listed by 2-digit abbreviation (Italy = “IT,” Mexico = “MX,” United States of America = “US,” Uruguay = “UY”)

Parasite lineage	Host species	Location	Publication
BNOW02 (*Haemo.*)	American barn owl (*Tyto furcata*)	California, US	Ishak et al. 2008
	American kestrel	Delaware, US	This paper
	American kestrel	Utah, US	This paper
BNOW03 (*Haemo.*)	American barn owl	California, US	Ishak et al. 2008
	American kestrel	US	Outlaw & Ricklefs 2009
	American kestrel	San Luis Potosi, MX	Tinajero et al. 2019
	American kestrel	Delaware, US	This paper
	American kestrel	Utah, US	This paper
FASPA02 (*Leuco.*)	American kestrel	California, US	Walther et al. 2016
	Merlin (*Falco columbarius*)	Livorno, IT	Nardoni et al. 2020
	American kestrel	Utah, US	This paper
FASPA03 (*Haemo.*)	American kestrel	San Luis Potosi, MX	Tinajero et al. 2019
	American kestrel	Utah, US	This paper
SALAUR01 (*Haemo.*)	Golden-billed saltator (*Saltator aurantiirostris*)	UY	Durrant et al. 2006
	American kestrel	US	Outlaw & Ricklefs 2009
	American kestrel	Delaware, US	This paper
	American kestrel	Utah, US	This paper

## Discussion

Little is known about parasite prevalence and diversity among distinct populations of American kestrels. We screened American kestrels from two geographic regions (Delaware and Utah) that presumably represent genetically distinct populations (Ruegg et al. 2021) and found that both groups harbor largely similar avian haemosporidian parasite prevalence and lineage diversity (Table 2). In fact, the lineages from Delaware and Utah have also been found in other parts of the kestrels’ range from other genetically distinct populations (Table 3). The most common parasites of kestrels in our study were *Haemoproteus* parasites, in keeping with previous studies (Dawson and Bortolotti 1999; Frixione and Rodríguez-Estrella 2023; Tinajero et al. 2019; Wiehn et al. 1997). The similarity of parasite lineages among these two host populations could be caused by several factors: (1) broadly distributed vectors that readily disperse among kestrel populations (or historically dynamic ranges of either vectors or kestrels), (2) alternative (and currently undetected) host species dispersing among kestrel populations, (3) transmission of the parasites during migration or on overwintering sites, or (4) cyt *b* lineages may not reflect true population genetic structure in the parasite (i.e., the parasite might exhibit distinct genetic populations at other genetic loci; but see Hellgren et al. 2021). Considering the first possibility, we know little about the range, distribution, and prevalence of vector species (particularly biting midges and black flies) that transmit haemosporidian parasites to North American birds, so considerable natural history research is needed to investigate this possibility. This may also explain why two of the kestrel lineages (BNOW02 and BNOW03) have been found in American barn owls (*Tyto furcata*); both host species nest in boxes or cavities and may share a vector that prefers nest boxes. The second option seems unlikely because the lineages infecting kestrels in this study are largely specialists of kestrels (Table 3) and it is not clear what alternative host would regularly move longitudinally across the USA to provide opportunity for parasite transmission between the populations. The third possibility is difficult to assess because we do not know the overwintering locations of kestrels from the Delaware and Utah populations (although this could be studied in the future by tracking kestrels). However, one of the *Haemoproteus* lineages (BNOW03) was found in juvenile birds in Delaware, indicating that transmission does occur on the breeding grounds. The fourth option is something to be investigated in the future. Nuclear genes evolve faster than mitochondrial genes in haemosporidians (Galen et al. 2018) and so might show evidence of population genetic structure not apparent in cyt *b* sequences (Hellgren et al. 2015; Huang et al. 2019). Genetic sequencing techniques like hybrid capture (Huang et al. 2018) and transcriptome sequencing (Galen et al. 2019) have recently facilitated sequencing avian haemosporidian nuclear DNA.

In the Delaware kestrel population, we found two juvenile birds and seven adult birds infected with the *Haemoproteus* parasite BNOW03, confirming that lineage is transmitted within the population during the breeding season. However, we do not know whether the haemosporidian lineages in this study are transmitted on the wintering grounds of kestrels. While we know kestrels can migrate as far south as Mexico (Goodrich and Smith 2008) and Nicaragua (Hunt et al. 2023), we do not know whether kestrels from Delaware and Utah migrate or overwinter in the same locations, although it has been suggested in Ruegg et al. (2021) that they do not overlap. Interestingly, the *Leucocytozoon* lineage FASPA02 found in Utah has been found in a close relative of the American kestrel, a merlin (*Falco columbarius*), in Italy (Nardoni et al. 2020) making it one of a few parasite lineages found on multiple continents that are not connected by north–south avian migratory flyways (Ellis et al. 2019). Nuclear DNA sequencing of this lineage on both continents could be used to determine the direction of parasite dispersal (Huang et al. 2019).

In Delaware, adult kestrels were more frequently infected with haemosporidian parasites than juveniles. These results are consistent with previous studies and likely reflect the fact that these infections can be long lasting (Latta and Ricklefs 2010). There was no significant difference in infection prevalence between male and female kestrels in both Delaware and Utah. This is an expected outcome given the results of previous studies (Ellis et al. 2020; Granthon and Williams 2017; Huang et al. 2020; Jing et al. 2023).

According to the MalAvi database (updated 2025–02–25 and including the data from this study), American kestrels have been found infected with six *Haemoproteus* lineages (BNOW02, BNOW03, FASPA01, FASPA03, FASPA04, SALAUR01), one *Leucocytozoon* lineage (FASPA02), and one *Plasmodium* lineage (MYCAME02). Among the *Haemoproteus* lineages, BNOW02 and BNOW03 have been found infecting American kestrels and barn owls, FASPA01, FASPA03, and FASP04 have only been found infecting American kestrels, and SALAUR01 was found infecting American kestrels and a passerine, the golden-billed saltator (*Saltator aurantiirostris*) in Uruguay. The *Leucocytozoon* lineage, FASPA02, has been found infecting American kestrels and a captive merlin (*Falco columbarius*) in Italy. Finally, the *Plasmodium* lineage, MYCAME02, is a generalist that has been found infecting 20 host species from seven taxonomic orders (Passeriformes, Ciconiiformes, Anseriformes, Phoenicopteriformes, Falconiformes, Strigiformes, and Cuculiformes) across North and South America. Therefore, American kestrels appear to be typically infected by specialized parasite lineages (i.e., parasites with one or a few host species; although SALAUR01 that was shown to infect a passerine is arguably more of a generalist despite infecting only two hosts), but are infected by at least one generalist (MYCAME02).

Parasite species and lineages can turnover across space as a result of host turnover or geographic barriers to dispersal. One way to study parasite lineage turnover while controlling for the effect of host turnover is to examine parasite distributions across populations of host species. With kestrels, there is likely limited gene flow across the continental United States (Ruegg et al. 2021); however, populations in Delaware and Utah harbor similar parasite lineages (Table 2) and are infected at similar rates. In fact, kestrels in general seem to harbor the same suite of haemosporidian parasites across their distribution. Further analysis of parasite genetics, vector ecology, and kestrel migration/overwintering are needed to clarify underlying mechanisms. Investigations across the regional distributions of host species are important to clarify the causes of parasite distributions and infection risk.

## Data Availability

All data are presented in the manuscript.
